# Pancreatic Panniculitis: A Rare Sign of a Common Disease

**DOI:** 10.7759/cureus.84042

**Published:** 2025-05-13

**Authors:** Catarina Assis, Antonio Gomes

**Affiliations:** 1 General Surgery, Hospital de Vila Franca de Xira, Unidade Local de Saúde (ULS) Estuário do Tejo, Vila Franca de Xira, PRT; 2 Surgery, Hospital Professor Doutor Fernando Fonseca, Unidade Local de Saúde (ULS) Amadora/Sintra, Amadora, PRT

**Keywords:** ductal pancreatic adenocarcinoma, pancreatic disease, pancreatic panniculitis, pancreatitis complication, panniculitis associated with pancreatic disease, rare panniculitis

## Abstract

Pancreatic panniculitis is a rare form of panniculitis that occurs in a minority of patients with pancreatic disease, including malignancy. We present the case of a 51-year-old male patient admitted to the hospital with acute gallstone pancreatitis. During his admission, he reported the appearance of painful subcutaneous erythematous nodules on his lower limbs. A skin biopsy was done, revealing the presence of “ghost adipocytes,” compatible with pancreatic panniculitis. Although the pathogenesis is unknown, it is thought to be associated with circulating pancreatic enzymes and clinically presents with subcutaneous erythematous to brownish nodules, mostly on the lower limbs, that typically resolve with the underlying disease. With the high prevalence of pancreatic disorders, it is very important that clinicians recognize associated skin lesions, as these may even be the presenting symptom of an underlying pancreatic pathology.

## Introduction

Pancreatic panniculitis is a rare type of panniculitis that occurs in 0.3%-3% of patients with pancreatic disease, mostly acute or chronic pancreatitis or pancreatic carcinoma (most commonly acinar cell carcinoma, but also with intraductal papillary mucinous neoplasm). Despite being rare, it may precede the clinical manifestation of the pancreatic disorder or even be the presenting symptom in up to 40% of cases. We present a case of pancreatic panniculitis associated with acute pancreatitis, as an opportunity to present a rare manifestation of a prevalent disorder [1-4].

## Case presentation

We present the case of a 51-year-old male patient who presented to the hospital complaining of upper abdominal pain, vomiting, and dark-colored urine. The symptoms had started the previous day. He had a recent admission due to acute calculous cholecystitis. His past medical history included obesity (BMI 33.7 kg/m^2^), obstructive sleep apnea treated with continuous positive airway pressure (CPAP), hypertension, deep vein thrombosis, and nephrolithiasis.

**Figure 1:**
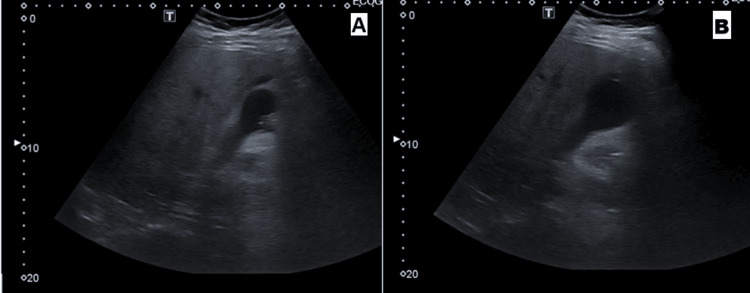
(A and B) US scan - revealing liver with normal dimensions, with steatosis but no nodular lesions. No dilation of intra- or extra-hepatic bile ducts. Gallbladder with lithiasis

On physical examination, he had a tender upper abdomen. His bloodwork on admission revealed raised aspartate transaminase (AST) 342 IU/L, alanine transaminase (ALT) 388 IU/L, and gamma-glutamyl transferase (GGT) 592 IU/L, hyperbilirubinemia 3.57 mg/dL, raised lactate dehydrogenase (LDH) 353 IU/L and amylase 1,592 IU/L, and raised CRP 14.11 mg/dL (Table 1). An US was done, which revealed a mild thickening of the gallbladder wall, with cholelithiasis but no associated biliary tree dilation (Figure 1).

**Table 1:** Laboratory results AST: aspartate transaminase; ALT: alanine transaminase; GGT: gamma-glutamyl transferase; LDH: lactate dehydrogenase

	Result	Reference range
AST	342 IU/L	15-37
ALT	388 IU/L	16-63
GGT	592 IU/L	15-85
Total bilirubin	3.57mg/dL	<1
Direct bilirubin	3.06 mg/dL	0-0.3
LDH	353 IU/L	85-227
Amylase	1,592 IU/L	25-115
CRP	14.11 mg/dL	0.05-1

**Figure 2:**
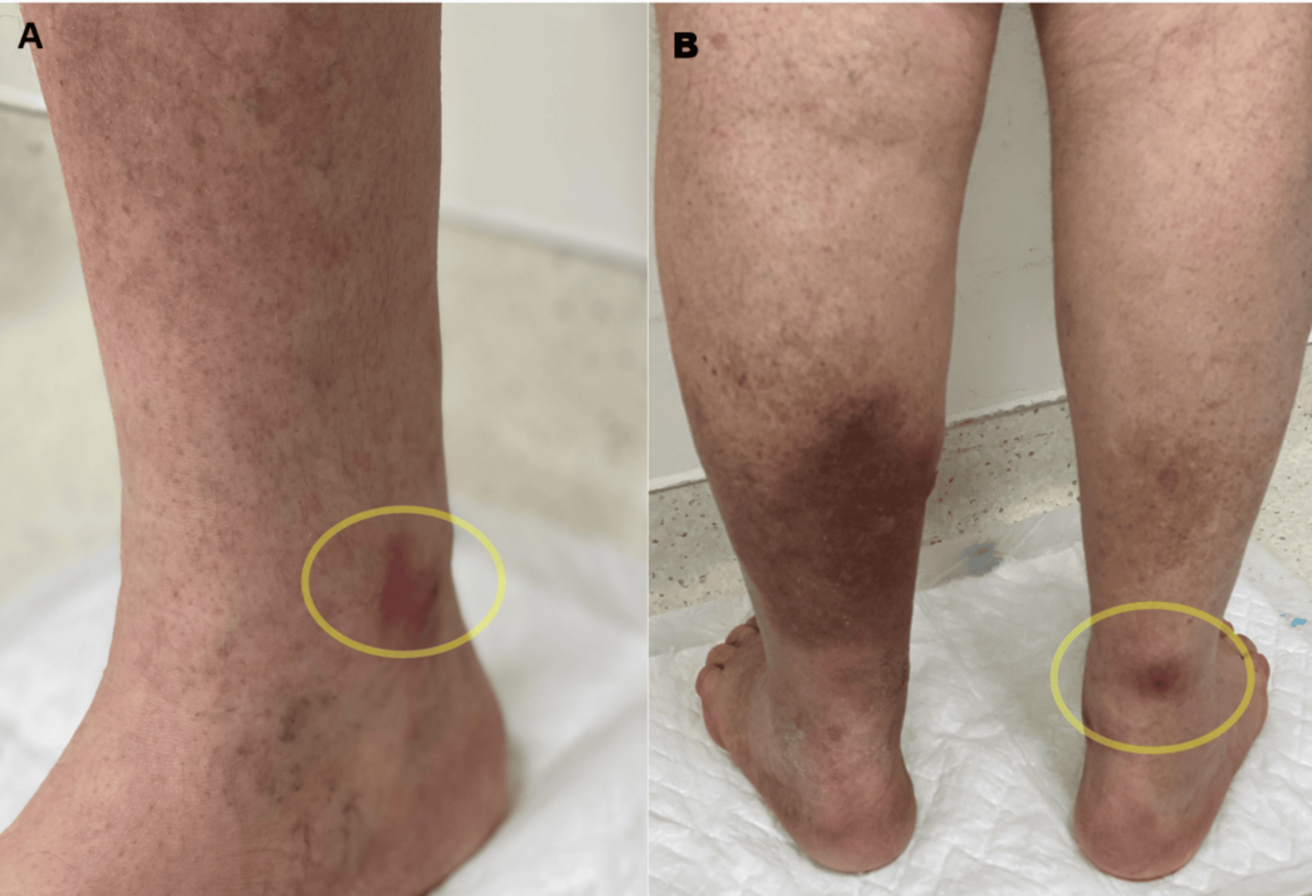
(A and B) Erythematous lesions on the patient’s lower limbs

**Figure 3:**
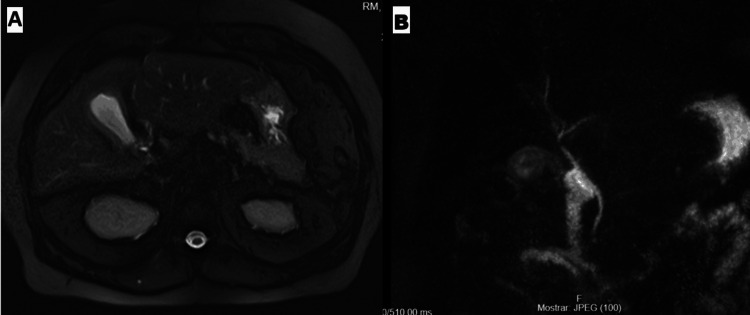
(A and B) MRCP: excluded choledocholithiasis but revealed gallbladder wall thickening MRCP: magnetic resonance cholangiopancreatography

The patient was admitted with the diagnosis of acute pancreatitis. After an initial improvement, on the fifth day of admission, the patient reported worsening pain associated with oral diet progression into a hypolipidemic diet and the appearance of two painful subcutaneous erythematous nodules (Figure 2): one on the internal malleolus, posterior leg region and another on the thigh, which were reviewed by a dermatology consultant, who raised the suspicion of pancreatic panniculitis. Lesion biopsies were done, and supportive treatment for the pancreatitis was continued. Magnetic resonance cholangiopancreatography (MRCP) (Figure 3) was done, which excluded choledocholithiasis but revealed worsened gallbladder wall thickening. The patient was then started on IV antibiotics for associated cholecystitis, with subsequent clinical and laboratory improvement. He was discharged on the 14th day of hospitalization.

**Figure 4:**
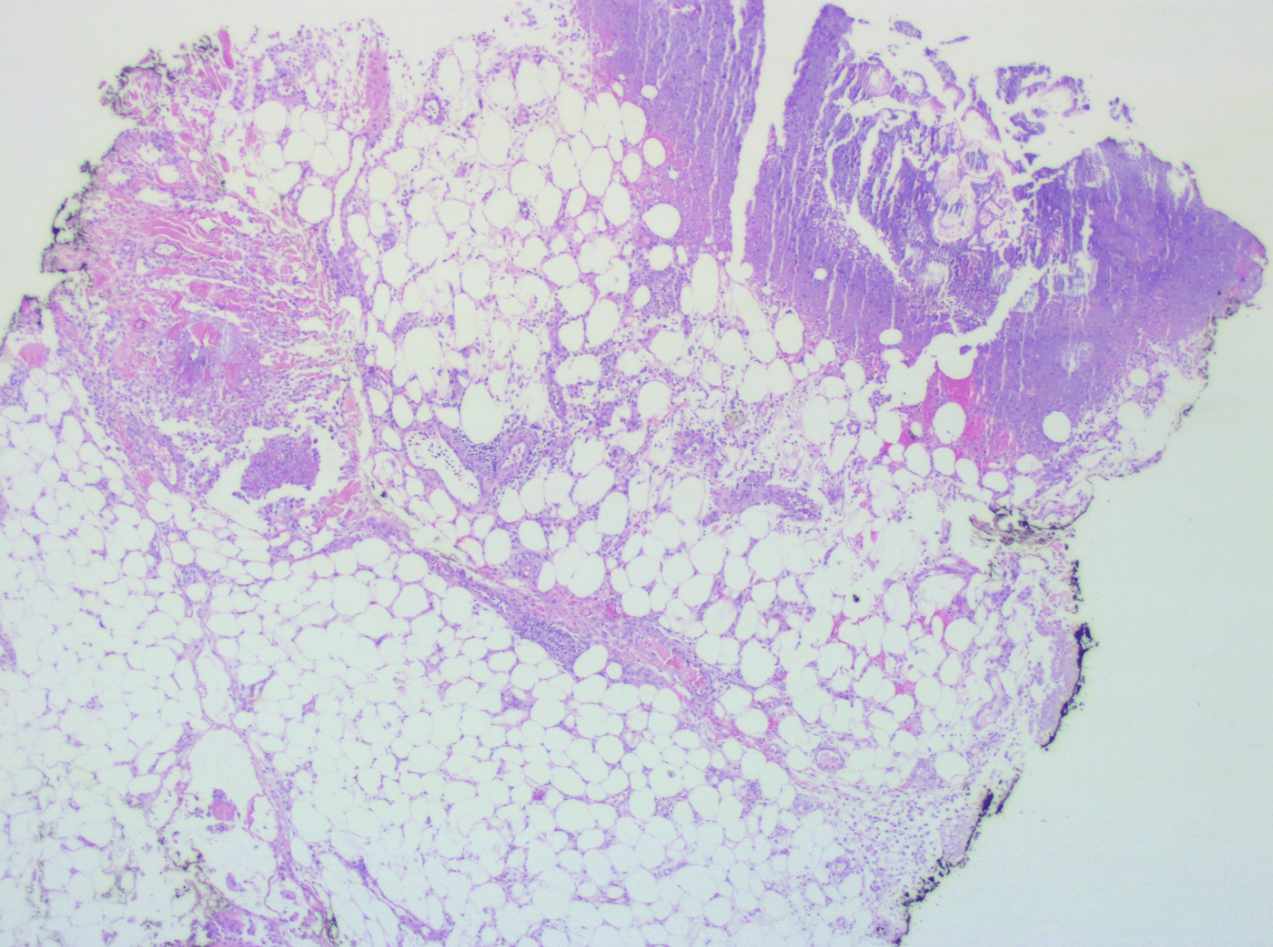
Lesion biopsies revealing the characteristic “ghost” adipocytes

The biopsy of the skin lesions revealed the presence of “ghost adipocytes,” focal calcium deposits (saponification), and a mixed inflammatory infiltrate with neutrophils and histiocytes, compatible with pancreatic panniculitis (Figure 4).

The patient was reviewed in an outpatient clinic three months following the discharge, with healed skin lesions. The patient refused elective cholecystectomy and remains symptom-free four years following the episode, following a strict low-fat diet.

## Discussion

Panniculitides are a group of conditions associated with inflammation of the subcutaneous fat [1]. They may be idiopathic, or secondary to trauma, cold, infection, systemic disorders such as lymphoproliferative disorders, connective tissue disorders, or even sarcoidosis. Pancreatic panniculitis is a rare type of panniculitis, which was primarily described by Chiari in 1883 and in the English literature in 1947 by Dahl et al. [2-8].

The pathogenesis is unknown, but it is thought that circulating trypsin increases capillary and lymphatic permeability, allowing the passage of amylase and lipase into the subcutaneous tissue, leading to adipocyte necrosis, fat hydrolysis, and saponification, which also leads to an inflammatory response. This theory is supported by evidence of amylase and lipase in the skin lesions [6].

The role of lipase is also supported by the presence of monoclonal antibodies anti-lipase in the necrotic tissue samples. Nevertheless, only a small percentage of patients with pancreatic disorders develop the condition, and there have been reported cases associated with normal blood levels of amylase and lipase; hence, there may be additional unknown factors [1,6,9].

Clinically, it presents with subcutaneous erythematous/brownish nodules, mostly on the lower limbs - peri-malleolar and pretibial, but may also present in thighs, glutes, dorsal, and abdomen. They may become ulcerated and release a brownish oily exudate (liquefied necrotic subcutaneous fat). The lesions typically heal after the resolution of the underlying disease.

There has also been a described involvement of fatty tissue in the gastrointestinal tract (GIT), bone marrow, and periarticular fat, which may be associated with the development of arthritis - pancreatitis, panniculitis, and polyarthritis (PPP) syndrome [1,2,6,9,10].

The histopathology was initially described by Szymanski and Bluefarb in 1961 - with an acute inflammatory infiltrate and the presence of the pathognomonic anucleated adipocytes “ghost cells,” whose membranes were partially digested by the pancreatic enzymes [11]. The lesions typically heal with fibrosis and lipoatrophy [1,2,7,12].

There is no specific treatment - the treatment is directed at the underlying disease and supportive care (Poelman and Nguyen). Chronic pancreatitis may present with relapsing lesions. PPP syndrome and disseminated fat necrosis are associated with a dismal prognosis [10,13].

## Conclusions

With the high prevalence of pancreatic disorders, it is very important that surgeons are aware of possible associated skin conditions. Furthermore, it is also important that dermatologists, internal medicine, and general practitioners recognize that pancreatic panniculitis may be the presenting symptom in a patient with an underlying pancreatic pathology and possibly an underlying pancreatic malignancy.
